# Kisspeptin and Metabolism: The Brain and Beyond

**DOI:** 10.3389/fendo.2018.00145

**Published:** 2018-04-16

**Authors:** Monika Dudek, Kamil Ziarniak, Joanna H. Sliwowska

**Affiliations:** Laboratory of Neurobiology, Institute of Zoology, Poznań University of Life Sciences, Poznań, Poland

**Keywords:** kisspeptin, obesity, diabetes, undernutrition, metabolism, reproduction

## Abstract

Apart from the well-established role of kisspeptin (Kp) in the regulation of reproductive functions, recent data described its action in the control of metabolism. Of particular interest for the review is the population of Kp neurons localized in the arcuate nucleus (ARC) of the hypothalamus, the site of the brain where reproductive and metabolic cross talk occurs. However, within the hypothalamus Kp does not work alone, but rather interacts with other neuropeptides, e.g., neurokinin B, dynorphin A, proopiomelanocortin, the cocaine- and amphetamine-regulated transcript, agouti-related peptide, and neuropeptide Y. Beyond the brain, Kp is expressed in peripheral tissues involved in metabolic functions. In this review, we will mainly focus on the local action of this peptide in peripheral organs such as the pancreas, liver, and the adipose tissue. We will concentrate on dysregulation of the Kp system in cases of metabolic imbalance, e.g., obesity and diabetes. Importantly, these patients besides metabolic health problems often suffer from disruptions of the reproductive system, manifested by abnormalities in menstrual cycles, premature child birth, miscarriages in women, decreased testosterone levels and spermatogenesis in men, hypogonadism, and infertility. We will review the evidence from animal models and clinical data indicating that Kp could serve as a promising agent with clinical applications in regulation of reproductive problems in individuals with obesity and diabetes. Finally, emerging data indicate a role of Kp in regulation of insulin secretion, potentially leading to development of further therapeutic uses of this peptide to treat metabolic problems in patients with these lifestyle diseases.

## Double Burden of Malnutrition as a Serious Worldwide Concern

A serious public health challenge facing many countries worldwide, recognized by the World Health Organization (WHO), is the double burden of malnutrition. It is characterized by the coexistence of contrasting forms of malnourishment: undernutrition along with overweight (1). Actions proposed by the WHO include interventions, programs and policies to reduce the risk or this burden. Understanding the neuroendocrine background for such conditions could lead to improvement of therapeutic strategies.

## Kisspeptin (Kp) as a Possible Link between Metabolism and Reproduction

All organisms need food to meet their functional requirements for immediate metabolic needs and conserve energy excess in the form of fat for times of food scarcity (2). Another crucial and energy demanding process, i.e., reproduction, is needed for the perpetuation of species (3). Kp, encoded by *KISS1* in the human and non-human primates and *Kiss1* in non-humans, its receptor *KISS1R* and *Kiss1r* or *GPR54*, respectively, are involved in the regulation of the hypothalamic–pituitary–gonadal (HPG) axis (4, 5). Importantly, energy metabolism is tuned to distinct sex-specific functions. Whereas in males metabolism may represent a default state, in females it is linked to specific requirements during gestation, parturition, and lactation (6). The arcuate nucleus (ARC) of the hypothalamus, expressing numerous neuropeptides including Kp, has been an area of focus for both circuits regulating metabolism and reproduction (7, 8).

## Role of Kp and Other Neuropeptides in Metabolism Control: Focus on the Brain

Extensive data support a major role of the ARC Kp neurons population in conveying information on metabolism to the gonadotropin-releasing hormone (GnRH) neurons, localized at the apex of the HPG axis (9). Disturbances in positive and negative energy balance often result in impairments of fertility, such as hypothalamic hypogonadism, frequently found in case of obesity and diabetes (10–12). However, Kp does not work alone, as Kp immunoreactive (-ir) neurons in the ARC also express neurokinin B (NKB) and dynorphin A (DYN A) and are termed KNDy neurons, and their degree of colocalization varies among species (13–18). NKB belongs to tachykinins, is encoded by the *Tac2* or *TAC3* gene (in rodents and humans, respectively), and works *via* the NK3R receptor [Tacr3 encoded by *Tacr3* and *TACR3*, animals and human genes, respectively (19)]. DYN A is an endogenous opioid (encoded by *pDyn* and *PDYN*, animal and human genes, respectively) working alongside the kappa-opioid receptor, encoded by the *Oprk1* in animals and *OPRK1* in humans genes (13, 20). KNDy neurons regulate the secretion of GnRH, while NKB stimulates and DYN A inhibits Kp (21–24).

Neuropeptide expression in KNDy neurons depends on the metabolic status. In chronically obese female mice a decrease was observed in *Kiss1* mRNA in the ARC (25), while fasting reduced *Kiss1* mRNA expression in the hypothalamus in rats and mice (10, 26, 27). In diabetic male rats, a decrease in *Kiss1* mRNA levels was found in the hypothalamus and a lack of an increase in *Kiss1* mRNA after gonadectomy [GDX (28)]. Finally, an increased number of Kp-ir neurons and an altered response after GDX in the ARC were reported in streptozotocin (STZ)-induced diabetic rats (11). Current studies in our laboratory are exploring the effects of obesity, diabetes and GDX in female rats (29).

**Figure 1:**
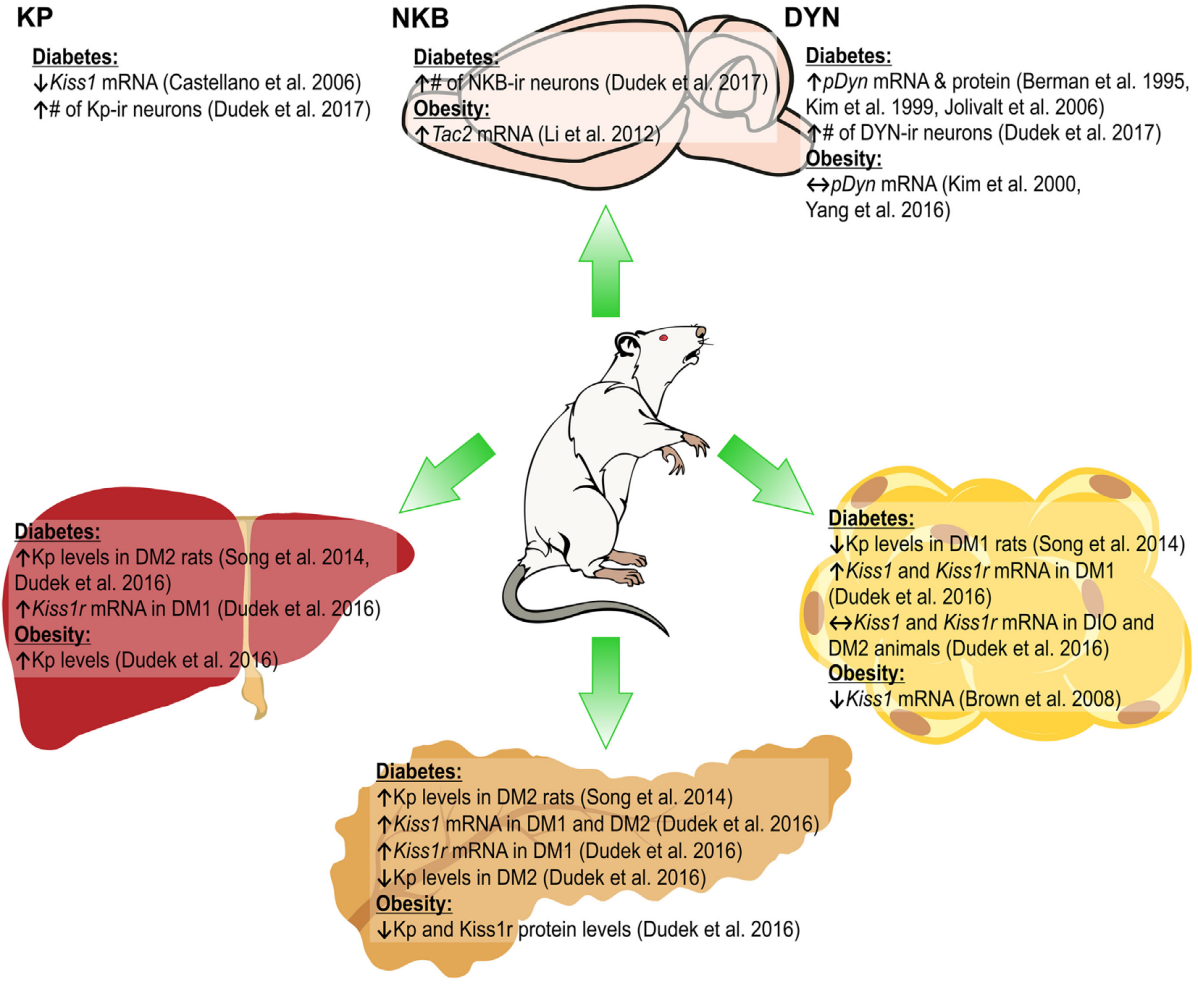
Changes in the expression of KNDy peptides [kisspeptin (Kp), neurokinin B (NKB), and dynorphin (DYN)] in the arcuate nucleus of the hypothalamus (ARC; upper part) and Kiss1/Kiss1r in peripheral organs (lower part) of rat due to diabetes obesity. ↑, increase; ↓, decrease; ↔, no effect (11, 26, 28, 30, 33–36, 54, 56).

Neurokinin B expression is also dependent on the metabolic status, as in pubertal female rats fed high-fat diet (HFD) higher expression of *Tac2* mRNA in the ARC was found (30). In db/db diabetic mice, *Tac2* mRNA levels increased in the hypothalamus (31), while in diabetic type 2 (DM2) male rats the number of NKB-ir neurons in the ARC increased (11). By contrast, under fasting conditions in female rats the expression of *Tacr3* and *Tac2* mRNA in the ARC decreased (19). The metabolic status was found to influence the DYN system, with an increase in DYN concentrations in the dorsomedial hypothalamic nucleus reported in obese ob/ob mice (32). In HFD-fed adult mice and obese rats, no changes were observed in prodynorphin (*proDyn*) expression in the ARC (33, 34). In STZ-induced diabetic rats, an increase in *proDyn* mRNA and protein level in the hypothalamus was shown (35, 36), while DM2 male rats had higher numbers of DYN A-ir in the ARC (11). Food restriction increased *proDyn* mRNA levels in the lateral hypothalamus (Figure 1) (37).

These results indicate effects of the metabolic status on the expression of KNDy peptides; however, they may depend on species, strains, sex of animals, as well as the experimental design, e.g., diet composition and duration or diabetes induction methods.

Besides KNDy neurons, in the ARC two other neuron populations may be identified as a link between metabolism and reproduction: cells expressing proopiomelanocortin (POMC) and the cocaine and amphetamine-regulated transcript (CART) and a population expressing agouti-related peptide (AgRP) and neuropeptide Y [NPY (38, 39)]. First, while POMC neurons are suggested to decrease food intake, and catabolic and anabolic effects of CART in the ARC and lateral hypothalamic area, respectively, were reported, NPY/AgRP cells play an important orexigenic role in energy homeostasis (38, 40–42). Second, POMC and NPY/AgRP neurons respond to insulin. Electrophysiological studies have shown that this hormone excites POMC and inhibits NPY/AgRP neurons (43, 44). Third, NPY/AgRP- and POMC-derived peptides, e.g., alpha-melanocyte stimulating hormone, directly influence GnRH neuron excitability (45). Fourth, anatomical data indicate a direct contact of GnRH cells with POMC/CART and NPY/AgRP neurons (8, 46, 47). Fifth, there is evidence that KNDy cells in the ARC are locally connected with AgRP and POMC neurons (48, 49). However, there are differences in the ARC neuropeptides coexpression in the monkey compared to rodents (8). Finally, Kp shows stimulatory effects on POMC and inhibitory effects on NPY neurons in the ARC (50). Thus, both POMC/CART and AgRP/NPY cell populations appear to be good candidates as GnRH regulators (51).

## Evidence for the Role of Kp in Metabolism Control: Beyond the Brain

The Kp gene and its receptor are expressed not only in the central nervous system (CNS) but also in the peripheral tissues (52, 53). In this review, we discuss its role in the pancreas, liver, and adipose tissue. In view of two extensive reviews on the subject (52, 53), we will focus mainly on the latest findings in the field.

Several lines of evidence indicate a role of Kp in metabolism regulation in the peripheral tissues (53, 54, 55). First, Kp and its receptor are expressed in peripheral tissues controlling metabolism (26, 54, 56). Second, at times of metabolic imbalance (e.g., undernutrition, obesity, and diabetes), alterations of the Kp system in these tissues were observed (57). Third, *in vitro* experiments indicate an action of Kp on lipid metabolism (55). Fourth, evidence for a function of Kp in metabolism control came from a study of *Kiss1r* KO animals. *Kiss1r* KO females had a greater body weight (BW), hyperinsulinemia, increased adiposity, elevated fasting basal glucose levels as well as impaired glucose tolerance (58). However, the observed obesity cases were not due to hyperphagia, but rather reduced metabolism. *Kiss1r* KO females had dramatically decreased energy expenditure. By contrast, *Kiss1r* KO males were characterized by normal BW and glucose tolerance. It would be of interest to study possible insulin resistance in this experimental model. Of particular relevance for this review, in the DM2 state insulin resistance is observed. Moreover, DM2 is often associated with obesity and accounts for roughly 90% of all diabetes cases (59). Adiposity, hyperinsulinemia and decreased metabolism were already seen at a younger age in *Kiss1r* KO females, with impaired glucose tolerance and feeding developing later in adulthood, after BW was significantly increased. Thus, an early life decrease in metabolism and energy expenditure may underline the later emergency of the obese phenotype of adult *Kiss1r* KO females (60). This phenotype could arise from defective signaling in the brain, and/or peripherally. Data on the expression of NPY, POMC, leptin receptor, ghrelin receptor, and melanocortin receptor 3 and 4 genes involved in the appetite regulating system of the hypothalamus generally showed no changes in the above-mentioned genes in *Kiss1r* KO mice with GDX. This suggests that obesity revealed in *Kiss1r* KO mice may reflect peripheral rather than central metabolic impairments (61). Below, we will update information on Kp in the peripheral tissues involved in control of metabolism.

## Kp, Pancreas, and Glucose Metabolism

**Figure 2:**
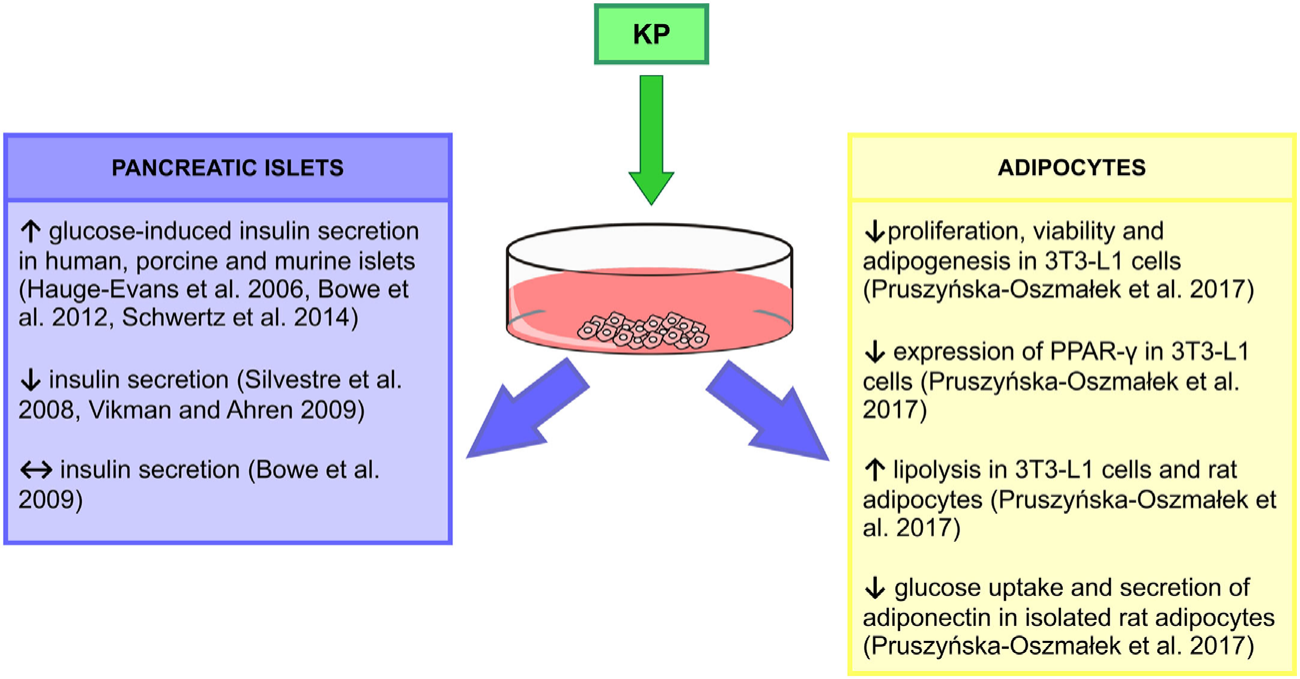
*In vitro* actions of kisspeptin (Kp). ↑, increase; ↓, decrease; ↔, no effect (55, 63, 65, 67–70).

Glucose homeostasis is regulated by insulin secreted from beta cells in the pancreatic islets (62). Moreover, insulin release is modulated by numerous G protein-coupled receptors including *Kiss1r* (63). Expression of the *Kiss1* gene in the pancreatic tissue was shown by Lee et al. (64), while later Hauge-Evans et al. (65) detected the expression of the Kp gene and its receptor in alpha and beta cells in human and murine islets, respectively. Both Kp and its receptor immunoreactivities colocalized with alpha and beta pancreas cells (65). Kisspeptin-54 (Kp-54) increased glucose-induced insulin secretion from human and murine islets, without any effect on basal secretion (65). Studies on rhesus monkeys (66) and rats (67) using kisspeptin-10 (Kp-10) confirmed *in vitro* observations. In addition, intracerebroventricular (icv.) administration of the peptide in rats had no effect on insulin levels, suggesting the peripheral site of action of Kp (67). Indeed, Kp-10 and kisspeptin-13 act directly at beta cells to potentiate insulin secretion stimulated by glucose in murine, porcine and human islets (63, 67, 68). Kp enhanced insulin secretion from murine islets in a dose-dependent manner through a G_βγ_-dependent pathway (63, 67). However, there are contradictory results indicating that Kp could inhibit (69, 70) or have no effect on insulin secretion (67), which may be related to the used doses and differences in protocols. Data from our laboratory confirmed expression of *Kiss1* and *Kiss1r* in the pancreas [mRNA and peptide (56)]. We have shown that diabetic type 1 (DM1) and DM2 male rats had increased pancreatic *Kiss1* mRNA levels. However, in the case of *Kiss1r*, an increase was recorded only in the DM1 group. By contrast, protein levels decreased in diet-induced obese (DIO) and DM2 animals. In addition, Kp and its receptor levels were undetected in the DM1 group (as a result of damage to pancreatic cells caused by STZ). Levels of Kiss1r protein were reduced only in the DIO group (56). Thus, in DM2/DM1 animals, the Kiss1/Kiss1r system may not function properly, thus being unable to control insulin levels (Figures 1 and 2).

## Kp in the Liver

The liver is an important organ, which acts as the body’s glucose reservoir (71). It is also responsible for maintaining steady circulating blood sugar levels by storing and producing glucose depending upon the body’s need (71). Only few studies focused on Kp in the liver. Kp-10 administered peripherally in male rats had antioxidant effects and increased the levels of free radical scavengers (superoxide dismutase and adenosine deaminase), suggesting protective effects on liver metabolism (72). Kp-10 in rats exposed to heat-induced oxidative stress decreased plasma corticosterone levels (73). Stressors enhance the activity of the hypothalamus–pituitary–adrenal axis, increasing corticosteroid levels. Since corticosterone may affect glucagon sensitivity and promote glycogenolysis, the authors hypothesized that glycogenolysis may be suppressed by the Kp-dependent reduction in corticosterone release (73). An extensive study conducted by Song et al. (54) revealed that in hyperglucagonemic diabetic animals (in HFD-induced diabetes and genetic leptin receptor-deficient db/db mice) liver Kp levels were increased. *In vitro* stimulation of mouse hepatocytes also increased *Kiss1* mRNA and protein levels. Moreover, DM2 patients exhibit increased liver and plasma Kp levels. In addition, we showed an increase in liver Kp expression in both DIO and DM2 male rats (56). Kp-10 and Kp-54 also impaired glucose tolerance, while selective liver Kp knock-down depressed glucose-sensitive insulin secretion (GSIS) and improved glucose tolerance and mice lacking liver Kiss1r exhibited improved glucose tolerance when fed HFD (54).

Based on the above findings, a tri-hormonal regulatory circuit between pancreatic alpha cells, hepatocytes, and beta cells was proposed, with Kp playing an important role in the liver to islet endocrine signaling. It was suggested that activation of the liver glucagon receptor stimulates insulin secretion by increased hepatic glucose production and hyperglycemia. However, liver glucagon action may also inhibit insulin secretion by stimulating Kp production. According to this model, in pancreatic beta cells in DM2 patients, the GSIS can be stimulated by hyperglycemia but inhibited by Kp. These findings extend a potential for Kp antagonism as a therapeutic tool to improve beta cell function in diabetic patients (Figure 2) (54).

## Kp in the Adipose Tissue

The major function of adipocytes is to store fat for periods of greater energy requirements (74). Control of these lipogenic and lipolytic processes is modulated by hormonal signals, with the adipose tissue emerging as an active participant in regulating physiologic and pathologic processes (75).

*Kiss1* mRNA was detected in rat (26, 56) and *KISS1* mRNA in human (76) adipose tissue and was shown to regulate the metabolic status. While fasting of rats increased adipose *Kiss1* mRNA expression in both sexes, a decrease was reported in *Kiss1* mRNA expression in adipose tissue of HFD and obese Zucker rats. These alterations were seen in visceral (epididymal) and subcutaneous (in the case of Zucker rats) adipose tissue (26). In women, a positive correlation between body mass index (BMI) and *KISS1* mRNA levels was found in the omental adipose tissue, but not in subcutaneous fat (76). *Kiss1* mRNA expression in adipose tissue is sensitive to sex steroids; while GDX in both sexes had no effect on *Kiss1* mRNA, supplementation of castrated animals with testosterone (males) and estradiol (females) increased *Kiss1* mRNA in rats (26).

We have revealed expression of mRNA and peptide for Kp in gonadal fat of male rats (56). While in DIO and DM2 animals expression of *Kiss1* and *Kiss1r* mRNA did not change, we were not able to detect peptides in these animals. By contrast, in DM1 rats there was a marked decrease in Kp and no change in *Kiss1r* levels. Our *in vitro* study showed that isolated rat adipocytes and mouse 3T3-L1 cells express mRNA and peptide for *Kiss1* and *Kiss1r* genes, and we found multiple actions of Kp-10 in these cells (55). First, Kp inhibited proliferation, viability, and adipogenesis in 3T3-L1 cells and decreased expression of PPAR-γ and CEBPβ—genes, involved in differentiation processes and adipogenesis. Second, Kp-10 increased lipolysis in rat adipocytes and 3T3-L1 cells by enhancing the expression of perilipin and hormone-sensitive lipase. Third, Kp-10 modulated lipogenesis. Finally, Kp-10 decreased glucose uptake and adiponectin secretion, while it stimulated leptin secretion from rat adipocytes (55). Thus, Kp could be a major factor in the regulation of adipocyte metabolism, at least *in vitro*. In particular, Kp-10 may slow the process of lipid accumulation *via* decreasing lipogenesis and increasing lipolysis. Further experiments are required to reveal the effects of Kp on adipose tissue *in vivo*. The stimulatory effect of Kp-10 on adiponectin, but not leptin levels was already shown in male rhesus monkeys, both fed and fasting (Figures 1 and 2) (66).

## Concluding Remarks and Future Directions

We reviewed data supporting the hypothesis that Kp is a link between metabolic cues and reproductive functions. On one hand, there have to be a proper amount of fat in the body to enter puberty, but on the other hand obesity can cause disturbances in puberty, followed by irregularities in testosterone production in men and ovulation in women (77, 78). Moreover, both puberty onset and obesity seems to be related to changes in Kp expression in the hypothalamus (7). However, peripheral Kp signaling may also play an important role in obesity or increasing body mass during adolescence. Within the hypothalamus, Kp does not act alone, but with other neuropeptides, e.g., NKB and DYN A, also being sensitive to the metabolic status. Moreover, relationships between the expression of Kp, NPY/AgRP, and POMC/CART were found. These neuropeptides have an impact on the hypothalamic GnRH functions, and complex interactions between these neurons in integration of metabolic and reproductive functions need to be revealed.

Although Kp is successfully used as a therapeutic target in human reproduction, including treatment of delayed and precocious puberty, subfertility, and contraception, much less is known on its role in the metabolic syndrome (79, 80). Kp-10 administered to men with DM2, and central hypogonadism enhanced endogenous testosterone secretion (81). Studies in animals indicate that Kp may be involved in the regulation of insulin secretion (54, 55, 63, 65, 69, 70). Recently, it was also revealed that Kp plasma concentration in the human is negatively correlated with BMI and waist circumference. Moreover, in non-diabetic individuals, Kp was shown to be associated with reduced glucose-stimulated insulin secretion (82). Further studies are needed to explore interactions between Kp and insulin in diabetic patients.

As most experiments are conducted on males and differences between sexes exist, there is a need to untangle alterations between females and males in the regulation of metabolic and reproductive functions. More research is required to understand interactions between Kp and metabolism in the peripheral tissues, where it can act in the paracrine manner. In addition, as tachykinins (neurokinins B and A, substance P) are extensively expressed within the CNS and at the periphery (83) and studies suggest a role of substance P in the regulation of metabolic functions (84, 85), it would be interesting to assess potential NKB/NK3R expression in peripheral tissues related to metabolism. More *in vivo* studies are needed on Kp action in the pancreas, liver, and fat tissue, while genetically modified animal models should be developed to elucidate mechanism(s) of its action. Basic scientists should participate in the WHO actions that have the potential to reduce the risk or burden of undernutrition and overweight by revealing the underlying mechanism(s). This may lead to the development of individually oriented therapeutic strategies against these lifestyle diseases.

## Author Contributions

Both MD and KZ participated in drafting, writing, and editing the manuscript. JHS participated in writing and editing the manuscript.

## Conflict of Interest Statement

The authors declare that the research was conducted in the absence of any commercial or financial relationships that could be construed as a potential conflict of interest.
